# Improved SVM-Based Soil-Moisture-Content Prediction Model for Tea Plantation

**DOI:** 10.3390/plants12122309

**Published:** 2023-06-14

**Authors:** Ying Huang

**Affiliations:** 1 Electronic Information School, Wuhan University, Wuhan 430072, China; huangying800816@163.com or hy@ltzy.edu.cn; 2 School of Automatic Control, Liuzhou Railway Vocational Technical College, Liuzhou 545616, China

**Keywords:** soil moisture prediction, hyper-parameter optimization, support vector machine

## Abstract

Accurate prediction of soil moisture content in tea plantations plays a crucial role in optimizing irrigation practices and improving crop productivity. Traditional methods for SMC prediction are difficult to implement due to high costs and labor requirements. While machine learning models have been applied, their performance is often limited by the lack of sufficient data. To address the challenges of inaccurate and inefficient soil moisture prediction in tea plantations and enhance predictive performance, an improved support-vector-machine- (SVM) based model was developed to predict the SMC in a tea plantation. The proposed model addresses several limitations of existing approaches by incorporating novel features and enhancing the SVM algorithm’s performance, which was improved with the Bald Eagle Search algorithm (BES) method for hyper-parameter optimization. The study utilized a comprehensive dataset comprising soil moisture measurements and relevant environmental variables collected from a tea plantation. Feature selection techniques were applied to identify the most informative variables, including rainfall, temperature, humidity, and soil type. The selected features were then used to train and optimize the SVM model. The proposed model was applied to prediction of soil water moisture in a tea plantation in Guangxi State-owned Fuhu Overseas Chinese Farm. Experimental results demonstrated the superior performance of the improved SVM model in predicting soil moisture content compared to traditional SVM approaches and other machine-learning algorithms. The model exhibited high accuracy, robustness, and generalization capabilities across different time periods and geographical locations with R^2^, MSE, and RMSE of 0.9435, 0.0194 and 0.1392, respectively, which helps to enhance the prediction performance, especially when limited real data are available. The proposed SVM-based model offers several advantages for tea plantation management. It provides timely and accurate soil moisture predictions, enabling farmers to make informed decisions regarding irrigation scheduling and water resource management. By optimizing irrigation practices, the model helps enhance tea crop yield, minimize water usage, and reduce environmental impact.

## 1. Introduction

Plant science encompasses a wide range of disciplines aimed at understanding the biology, ecology, and interactions of plants. Over the years, advancements in technology have provided researchers with new tools and techniques to explore the intricate world of plants. In recent times, machine learning (ML) and deep learning (DL) have emerged as powerful computational approaches with the potential to transform plant science research.

ML and DL are subsets of artificial intelligence (AI) that involve the development of algorithms and models capable of learning from and making predictions or decisions based on data. ML algorithms use statistical techniques to enable computers to learn patterns and relationships in data without being explicitly programmed, while DL, inspired by the structure of the human brain, utilizes artificial neural networks to model complex patterns and hierarchies in data.

The availability of large-scale datasets, coupled with advancements in computational power and algorithmic improvements, has made ML and DL highly effective in various fields. In plant science, these techniques have shown great potential in addressing challenges related to plant phenotyping, disease diagnosis, yield prediction, genomics, and crop improvement.

Sunny et al. proposed a new method for plant leaf recognition based on CapsNet, which consists of four parts: image sampling, image processing, feature extraction, and feature recognition [1].

Horng et al. proposed a harvesting system based on the Internet of Things technology and smart image recognition. The method uses an intelligent recognition model trained by a neural network, and then the system confirms whether the crop can be harvested, and finally uses a robotic arm to harvest the crop [2].

Disease diagnosis and pest management are critical aspects of plant science, as they directly impact crop health and productivity. Disease detection is conducted by experts using the naked eye, which is both time-consuming and troublesome [3].

ML and DL algorithms can analyze various data sources, including images, genomic data, and environmental parameters, to detect and classify plant diseases, assess disease severity, and optimize pest control strategies. These techniques provide valuable insights for early detection and timely intervention, leading to improved disease-management practices. K-Means [4], SVM [5], neural network [5], and Otsu’s thresholding algorithm [6] are the methods most used to detect and classify disease based on the features extracted from the image.

Convolution Neural Network Approaches (CNNS) is performed to select the dataset for training. Some architectures such as AlexNet [7,8], GoogLeNet [7,8], Inception v3 [9], CafeNet [10], and deep meta-architectures [11] are used to detect plant disease based on factors such as error rate, accuracy, and training time.

Van Dijk et al. summarized the application of machine learning in the context of plant science and plant breeding, which offers a suite of methods that enable researchers to find meaningful patterns in relevant plant data [12].

Esami et al. discussed and highlighted the application of AI-OA models in different steps of plant tissue culture, which provides a new view for future study objectives [13].

Singh et al. gave an overview of machine learning in the field of plant stress phenotyping for identification, classification, quantification, and prediction, which enable further exploration of these tools for facilitating practical use in plant breeding [14].

Jafari et al. presented a modeling and predicting morphological response of citrus to drought stress with different Artificial Neural Networks (ANNs), including Generalized Regression Neural Network (GRNN), Radial Basis Function (RBF), and Multilayer Perceptron (MLP), which are considered to be an effective tool for predicting plant morphological and physiological responses to drought stress [15].

Hesami et al. provided in-depth insight into plant system biology using machine learning, which is a good solution for studying plant system biology [16].

Grinblat and colleagues introduced a plant identification approach that concentrates on the vein patterns of leaves. They utilized deep convolutional neural networks to improve the recognition accuracy of legume species. This technique was reported to enhance the current state-of the art [17].

Mishra et al. proposed a hybrid approach in plant immune systems using Machine Learning, which provides new insights for understanding plant–microbe interactions [18].

Soil moisture content is one of the key parameters of plant growth. Machine learning and deep learning have also shown advantages in predicting soil water content. Soil moisture vastly affects tea growth, yield, and quality. Variations in soil moisture conditions within tea plantations directly affect the fertility of tea trees, and as a result, tea yield and quality. Appropriate soil moisture enhances tea yield and quality. Conversely, insufficient moisture retards tea tree growth, causes diseases, and even halts growth. Optimal soil moisture not only improves the root system’s capacity for nutrient absorption and utilization but also improves soil nutrient exploitation [19]. Therefore, precise soil moisture monitoring and management are imperative farming practices for high yield and excellent quality tea. Technological advances such as moisture sensors and computer modeling tools present constructive solutions for more effective irrigation management in tea plantations.

Various modeling techniques have been applied to predict SMC, including multiple linear regression (MLR), artificial neural network (ANN) models, and support vector machines (SVM) [20]. Among these techniques, SVM has shown superior performance due to its ability to handle small datasets and nonlinear complex relationships [21]. In addition, many scholars have proposed some solutions in soil water content prediction methods. Zhang, X., et al. presented that Conventional soil moisture measurement techniques like the weighing method, the tensiometer method, the resistance method, the neutron method, the standing wave ratio method, and the time domain reflection method are limited in measurement depth and scale [22]. 

Xu Qiao et al. proposes a soil moisture prediction approach based on multiple regression analysis to determine influential GPR indicators and radial basis function (RBF) neural network modeling using these indicators and measured soil moisture content [23]. Eftychia Taktikou et al. evaluates the ability of remotely sensed soil moisture to indicate in situ soil and canopy water content at fine spatial and temporal scales. Daily MODIS NDVI and diurnal land surface temperature difference were used to retrieve daily soil moisture, which was compared with in situ measurements; the results show the retrieved soil moisture can satisfactorily predict measured soil moisture and inform irrigation management [24]. Sankhadeep Chatterjee et al. developed a rapid, non-contact machine vision and AI-based method to estimate soil moisture from a single image. Three soil textures and organic matter levels were tested. Color features were extracted and used in ANFIS and multiple regression models to predict soil moisture, allowing soil moisture estimation from a single image [25]. Liang-hong C et al. used discrete wavelet transformation to decompose spectra, competitive adaptive reweighted sampling (CARS) to select key wavelengths, and partial least squares regression (PLSR) to model SMC, and then ELM to construct a nonlinear model for soil water content prediction, which provided an accurate SMC estimation model for arid soils [26]. Prakash S et al. used multiple linear regression, support vector regression and recurrent neural networks for prediction of soil moisture, which provided a good performance [27]. Data mining techniques and multiple linear regression were used to estimate tea yield analysis of four regions of Assam [28]. A multiple regression model was used to predict the average soil water content in the root zone, using soil water content at 5 cm depth, effective rainfall, and evapotranspiration as predictive variables [29]. L Esmaeelnejad et.al applied Multiple Linear Regression (MLR), Artificial Neural Network (ANN) and the Rosetta model to predict soil moisture [30]. T Aslan used SVR derived by Chaotic approach for the short term forecast of the soil water content [31]. Rf A et al. presented water management parameters prediction for irrigation management with RF (random forest), cubist (cubist regression), and GBM (gradient boosting machine) [32]. GAO Peng et.al establish prediction models of soil moisture content with long short-term memory (LSTM) and general regression neural network (GRNN), which has high accuracy and can be helpful for guiding irrigation and fertilization management [33].

Although the aforementioned method can improve the precision of soil moisture content assessment, it has limitations that need consideration.

The effectiveness of machine learning models depends on an adequate amount of high-quality training data. The caliber of the training data significantly resonates with the prediction outcomes of the model. Frequently, the training data may not satisfy the demands of machine learning models due to lack of information. Since inadequate training data can prevent models from precisely distinguishing distributional roles of data, it can cause poor generalization, overfitting, and unsatisfactory prediction outcomes.

Moreover, hyper-parameters’ choices in machine learning models significantly affect prediction outcomes. Incorrect hyper-parameter selection, such as learning rate, regularization coefficient, neural network number, network depth, etc., can lead to unsatisfactory prediction outcomes. The hyper-parameter selection remains an arduous and crucial concern in machine learning studies.

Therefore, this research aims to: 1. Develop an improved SVM model by incorporating meteorological factors for SMC prediction in tea plantations; 2. Investigate the use of population intelligence optimization algorithms to optimize machine learning hyper-parameters; and 3. Compare the performance of the improved SVM model with other existing models.

## 2. Research Object

### 2.1. Study Area Description

The Liucheng County is strategically situated in the subtropical monsoon zone of Guangxi, China—an area known for its remarkable climatic conditions. The county enjoys moderate temperatures and abundant light and rainfall, which are favorable for plant growth. However, the rainfall distribution across seasons tends to be uneven, leading to occasional seasonal water shortages. With an annual rainfall of 1300–1500 mm and an average temperature of 20 °C, the climate of the area is well-suited for the cultivation of various plants. The research area for this paper is the 58.4-hectare tea plantation located in Guangxi State-owned Fuhu Overseas Chinese Farm (N 109°21′, E 24°82′). The plantation is situated within Liucheng County, Guangxi, a region characterized by a subtropical humid climate. The area experiences an annual average temperature of 20.4 °C, with the hottest month having an average temperature of 28.2 °C. The annual average relative humidity is 77%, and the average annual sunshine duration is 1820 h. Despite adequate light, drought conditions still arise occasionally, particularly between March and May, due to irregular temporal rainfall distribution [34].

### 2.2. Soil Information Data System (SIDS)

To collect soil data from the tea plantation for this study, soil temperature sensors, soil moisture sensors, and soil electrical conductivity sensors were installed within the plantation. However, tea plantations are usually situated in hilly or sloping regions, which can create obstacles that affect wireless sensor node communication. To address this issue, wireless transmission and direct current (DC) line communication technology were utilized in tandem for dual data transmission. For this study, MEC20 sensors, manufactured by Dalian Zheqin Technology Co., were used to collect soil temperature, soil moisture content (SMC), and soil electrical conductivity (SEC) data. The sensors were selected based on their voltage output, cable length, and measuring range of SMC and SEC, all of which satisfy the necessary data collection requirements for the tea plantation. Voltage output sensors with a 6 m cable were chosen based on the measuring range of 0–100% for SMC and 0–5000 μS/cm for SEC. To ensure comprehensive and effective monitoring, the number and deployment of sensors were based on the actual plantation area. To adequately cover the Liuzhou tea plantation’s actual area, five sensor nodes were strategically deployed in a distributed pattern, with each node responsible for obtaining soil indicators for its assigned subarea.

First, divide the tea plantation into smaller zones based on factors such as topography, soil type, and any other relevant characteristics that may affect soil conditions. The number of zones will depend on the variability of the plantation.

Secondly, select sensor locations: Identify key representative locations within each zone where soil conditions can be measured effectively. The goal is to capture the spatial variability within each zone while minimizing the number of sensors. Consider factors such as slope, elevation, soil texture, and known variations in soil properties.

Lastly, decide on the depth at which you want to measure soil conditions. To monitor soil moisture in the root zone of tea trees accurately, sensors were buried at a depth of 10–15 cm. The suggestion of using sensors at varying depths enables the average soil moisture conditions to be obtained at different soil layers in the root zone [34].

It’s important to note that while five sensors provide valuable information, they may not capture the entire complexity of soil conditions across the entire tea plantation. However, this approach can help identify trends, variations, and patterns within the selected zones, guiding management decisions related to irrigation, fertilizer application, and overall soil health maintenance.

In addition, an integrated field weather monitor provided by Guangzhou Hairui Intelligent Technology Co., Ltd. is also installed, which is responsible for collecting environmental parameters related to this project, mainly including natural environmental parameters such as air temperature, air humidity, light intensity, rainfall, air pressure, wind speed, and wind direction [19].

## 3. Methods

**Figure 1:**
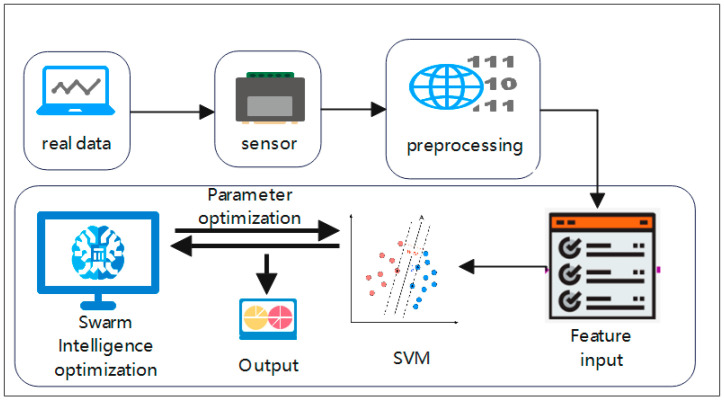
Proposed System.

The proposed model is presented in Figure 1.

Figure 1 shows the proposed model, which consists of sensors, data processing, feature input, support vector machine, swarm intelligence optimization, and result output. Firstly, the sensor deployed in the monitored area is used to collect data from the monitored area, and after data processing, the features are extracted and input to the crowd intelligence algorithm optimization after the support vector machine model is optimized to predict and obtain the prediction results. Data inputs include air temperature, relative humidity, rainfall, soil temperature and soil electrical conductivity, and the output of the data is soil water moisture.

### 3.1. Support Vector Machines

Support Vector Machines (SVMs) are a type of supervised machine learning algorithm utilized for classification and regression tasks. SVMs construct optimal hyperplanes that separate classes while simultaneously maximizing margins. With the application of kernel tricks, SVMs can handle complex, nonlinear problems and adapt to various classification tasks [19]. As a result, they are highly accurate and do not suffer from overfitting issues. Consequently, SVMs are an adaptable tool for many classification scenarios, providing precise solutions with minimal overfitting. To put it concisely, SVMs are a versatile and accurate machine learning algorithm used for classification and regression tasks.

Assume that the input sample is defined as Equation (1).

(1) $$D=\left\{\left(x_{1},y_{1}\right),\left(x_{1},y_{1}\right),\cdot \cdot \cdot ,\left(x_{1},y_{1}\right)\right\}\begin{array}{cc} & y_{i} \end{array}\in R$$

Therefore, the goal is to find a function f(x) whose value is as close to y as possible, defined as in Equation (2).

(2) $$f\left(x\right)=w^{T}x+b$$

The weight and bias values of the SVM model are represented by the variables “w” and “*b*”, respectively.

Equations (3) and (4) express the optimization problem:

(3) $$\underset{w,b}{\min}\frac{1}{2}{\left\|w\right\|}^{2}+C\sum_{i=1}^{m}\left(\xi_{i}+{\overset{}{\hat{\xi }}}_{i}\right)$$

(4) $$st.\left\{ \begin{array}{l} f\left(x_{i}\right)-y_{i}\leq \varepsilon +\xi_{i}\\ y_{i}-f\left(x_{i}\right)\leq \varepsilon +{\hat{\xi }}_{i}\\ \xi_{i}\geq 0,{\hat{\xi }}_{i}\geq 0,\begin{array}{cc} & i=1,2,\cdot \cdot \cdot ,m \end{array} \end{array}\right.$$

where C represents the penalty factor, while $\xi_{i}$ and ${\hat{\xi }}_{i}$ represent the introduced relaxation in the problem.

Equation (5) illustrates the pairwise problem from the above-mentioned problem

(5) $$\begin{array}{l} \mathrm{Max}\left[-\frac{1}{2}\sum_{i,j=1}^{m}\left(a_{i}-{\hat{a}}_{i}\right)\left(a_{j}-{\hat{a}}_{j}\right)K\left(x_{i},x_{j}\right)-\varepsilon \sum_{i=1}^{m}\left(a_{i}+{\hat{a}}_{i}\right)+\sum_{i=1}^{m}y_{i}\left(a_{i}-{\hat{a}}_{i}\right)\right]\\ s.t\{\sum_{i=1}^{m}\left(a_{i}-{\hat{a}}_{i}\right)=0,0\leq a_{i}\leq c,0\leq {\hat{a}}_{i}\leq c \end{array}$$

where $a_{i}$ and ${\hat{a}}_{j}$ are Lagrangian multipliers. The Lagrangian multipliers are represented by the variables $a_{i}$ and ${\hat{a}}_{j}$, respectively. $K\left(x_{i},x_{j}\right)$ is a kernel function.

Suppose the optimal solution is $a=\left[a_{1},a_{2},\cdots ,a_{m}\right]$, $\hat{a}=\left[{\hat{a}}_{1},{\hat{a}}_{2},\cdots ,{\hat{a}}_{m}\right]$. By adjusting the optimal solution, $w^{*}$ and $b^{*}$ are defined as Equations (6) and (7)

(6) $$\begin{array}{c} b^{*}=\frac{1}{N_{n}}\left\{\sum_{0<\alpha_{i}<C}\;\left[y_{i}-\sum_{x_{i}}\;\left(\alpha_{i}-\alpha_{i}^{*}\right)K\left(x_{i},x_{j}\right)-\varepsilon \right]+\right.\\ \left.\sum_{0<\alpha_{i}<CC}\;\left[y_{i}-\sum_{x_{i}}\;\left(\alpha_{i}-\alpha_{i}^{*}\right)K\left(x_{i},x_{j}\right)+\varepsilon \right]\right\} \end{array}$$

(7) $$w^{*}=\sum_{i=1}^{m}\left(a_{i}-{\hat{a}}_{i}\right)\varphi \left(x_{i}\right)$$

where $N_{n}$ is sample data of SVM.

Therefore, the decision function for SVR is defined as Equation (8).

(8) $$f\left(x\right)=w^{*}\cdot x+b^{*}=\sum_{i=1}^{m}\left(a_{i}-{\hat{a}}_{i}\right)\varphi \left(x_{i}\right)\varphi \left(x_{j}\right)+b^{*}=\sum_{i=1}^{m}\left(a_{i}-{\hat{a}}_{i}\right)K\left(x_{i},x_{j}\right)+b^{*}$$

The performance of the SVR model depends on the degree of match of the kernel function and the selection of the C value. Proper selection of the kernel function can effectively deal with nonlinear regression problems. However, if the value of C is too large, overfitting can easily occur, which reduces the generalization ability of the model. On the other hand, if the value of C is too small, underfitting occurs, and the model’s performance deteriorates. The selection of the C value achieves a balance between controlling the complexity of the model and generalization. Both factors determine the effectiveness of SVR. Therefore, optimizing these two parameters is necessary to obtain the best performance.

### 3.2. Bald Eagle Search Algorithm

The Bald Eagle Search algorithm, commonly known as BES, is renowned for its exceptional ability to conduct a global search and its effectiveness in solving multifaceted numerical optimization problems. An algorithm that has powerful global search capabilities can effectively solve various complex numerical optimization problems. The algorithm comprises of three phases, namely the selection, search, and swooping phases [19,35,36,37].

During the selection phase, the bald eagle initiates the optimization process by randomly selecting an area and evaluating the prey population to determine the best possible position to locate prey. The primary factors considered in determining the bald eagle’s position at this stage are experience and position change parameters (refer to Equation (9)).

(9) $$P_{i,new}=P_{best}+\alpha \times \gamma \times (P_{mean}-P_{i})$$

The parameter α signifies the control for changing the position and has a value between 1.5 and 2. The variable γ represents a random number ranging from 0 to 1. During the optimization process, $P_{best}$ denotes the bald eagle’s previously identified best search position, $P_{mean}$ indicates the prominence of information derived from the previous points, and $P_{i}$ refers to the i-th bald eagle’s position.

During the search phase, the bald eagle intensifies its search for potential prey to expedite the optimization process in the form of a spiral fly and identify the optimal swooping location. The mathematical model of spiral flight is represented by polar equations. The key parameters in the polar equation are polar angle $\theta \left(i\right)$ and polar diameter $r\left(i\right)$. Equation (10) plays a crucial role in this phase by determining the most suitable location for the eagle based on various parameters.

(10) $$P_{i,new}=P_{i}+x\left(i\right)\times \left(P_{i}-P_{mean}\right)+y\left(i\right)\times \left(P_{i}-P_{i+1}\right)\;x\left(i\right)=\frac{xr\left(i\right)}{\max\left(\left|xr\right|\right)}\;y\left(i\right)=\frac{yr\left(i\right)}{\max\left(\left|yr\right|\right)}\;\mathrm{xr}\left(i\right)=r\left(i\right)\times \sin\left(\theta \left(i\right)\right)\;\mathrm{yr}\left(i\right)=r\left(i\right)\times \cos\left(\theta \left(i\right)\right)\;r\left(i\right)=\theta \left(i\right)+R\times rand\;\theta \left(i\right)=a\times \pi \times rand$$

In the above equation, $\theta \left(i\right)$ and $r\left(i\right)$ represent the polar angle and polar diameter of the spiral equation, respectively. The parameter φ determines the angle between the search point and the central point, taking a value between 5 and 10. The parameter R determines the number of search cycles and takes a value between 0.5 and 2. *Rand* is a random number ranging from 0 to 1. Additionally, $x\left(i\right)$ and $y\left(i\right)$ indicate the bald eagle’s position in polar coordinates, and $P_{i+1}$ refers to the i-th bald eagle’s next updated position.

During the swooping stage, bald eagles swiftly swoop from the best spots in the search space towards the prey, while other species move towards the optimal spots and execute their attack on the prey. The position of the bald eagle during this stage is evaluated using Equation (11).

(11) $$P_{i,new}=\mathrm{rand}\times P_{best}+x1\left(i\right)\times \left(P_{i}-c1\times P_{mean}\right)+y1\left(i\right)\times \left(P_{i}-c2\times P_{best}\right)\;x1\left(i\right)=\frac{xr\left(i\right)}{\max\left(\left|xr\right|\right)}\;y1\left(i\right)=\frac{yr\left(i\right)}{\max\left(\left|yr\right|\right)}\;\mathrm{xr}\left(i\right)=r\left(i\right)\times \sin h\left(\theta \left(i\right)\right)\;\mathrm{yr}\left(i\right)=r\left(i\right)\times \cos h\left(\theta \left(i\right)\right)\;\theta \left(i\right)=a\times \pi \times rand\begin{array}{cc} & r\left(i\right) \end{array}=\theta \left(i\right)$$

The variables c1 and c2 denote the bald eagle’s exercise intensity concerning the optimal and central positions. The exercise intensity can have a value ranging between 1 and 2.

### 3.3. Modifications Bald Eagle Search Algorithm (MBESMBES)

In order to improve the convergence and convergence rate of BES during the optimization process, two optimizations have been made to the BES algorithm. The first optimization is for the initialization of the BES population positions, while the second is for the update of positions during the prey process.

(1) Optimization of Population Initialization Positions

The initialization of population positions is an important step towards swarm intelligence algorithms. The quality of the initialized positions has a significant effect on the convergence, convergence rate, and solution quality of the genetic algorithm. If the initial population positions are too centralized, the algorithm is prone to getting stuck in local optima, causing a decrease in accuracy at the cost of faster convergence. On the other hand, if the initial population positions are too dispersed, it is difficult for individuals to exchange information, making it challenging to find the global optimum direction, and requiring more time to find the optimal solution. This phenomenon can affect the convergence and convergence rate. Therefore, the choice of initial population positions should consider both convergence and convergence rate. Reasonable initialized population positions can balance the convergence rate with accuracy to find the global optimum direction gradually and achieve the optimal convergence effect. The more evenly distributed the population positions, the higher the coverage rate of the area, increasing the likelihood of finding optimal solutions. This enhances the probability of finding the optimal solution and speeds up the algorithm’s convergence.

In order to evenly distribute the initialization position of the vulture in the search area, the Tent chaotic mapping function is introduced to adjust the vulture group to enhance the diversification of the vulture group, avoid repetition, and help improve the global search ability of the algorithm. The introduction of random numbers can solve the problem of tent mapping in small cycle periods and fixed points. The mathematical expression for improving the tent map is shown in Equation (12)

(12) $$z_{n+1}=\left\{\begin{array}{c} 2z_{n}+rand/N\;\;\;0\leq z_{n}<0.5\\ 2\left(1-z_{n}\right)+rand/N\;0.5\leq z_{n}\leq 1 \end{array}\right.$$

where *rand* is a random number of the [0, 1] range, z represents the iteration value, and N is the total number of particles in the chaotic sequence.

For the specified search area, when the number of optimization parameters has only one parameter, the initialization position is shown in the Formula (13)

(13) $$Positon=L_{b}+z(U_{b}-L_{b})$$

where $L_{b}$ is the lower limit of the search area, and $U_{b}$ is the upper limit of the search area.

When the number of optimized parameters is two or more, the initialization of parameters is shown in Equation (14).

(14) $$Positon=X_{lb}+z.*(X_{ub}-X_{lb})$$

where

(15) $${X_{lb}=repmat(L}_{b,}SearchAgents\_no,1)$$

(16) $${X_{ub}=repmat(U}_{b,}SearchAgents\_no,1)$$

(2) Optimization of optimal position update

When the vulture selects the search area in the selection stage, it uses a random number γ to perform a spatial search, and the global search ability is limited. To solve this problem, the Fuch chaos map in the chaos map is introduced to optimize the random number γ. Fuch chaotic mapping can balance the search area, convergence is fast, and is not sensitive to initial values. Considering that the Fuchs chaos map generates negative numbers, the resulting chaotic sequences must be processed with absolute values. The specific definition is shown in Equation (17)

(17) $$f\left(i\right)=\left|cos\frac{1}{{f\left(i-1\right)}^{2}}\right|$$

where f (1) is a random number of [0, 1].

The update of the position of vultures in the hunting stage has a certain influence on the global optimal position and the average distribution position of all vulture groups. Therefore, a weight parameter ω is introduced, defined in Equation (18).

(18) $$\omega =\left\{\begin{array}{c} 0.9\cos\left(\frac{\pi }{2}\cdot \left(1-\frac{t}{T}\right)\right)\cdot {\left(\frac{fit\left(i\right)-GBest}{avg\left(fit\right)-GBest}\right)}^{\frac{t}{T}}\;fit\left(i\right)<avg\left(fit\right)\\ 0.9\cos\left(\frac{\pi }{2}\cdot \left(1-\frac{t}{T}\right)\right)\;fitness\left(i\right)<avg\left(fit\right) \end{array}\right.$$

From the characteristics of the cos function, it is known that at first the weight is small, the optimization speed is fast, and with the increase of the number of weights, the optimization speed is slower, so as to have a good convergence balance. At the same time, considering the individual adaptation value of the vulture, the weight value is small for the less adaptation value, and the weight value of the large adaptation value is large, which ensures that the vulture has a good performance to approach the search area.

Thus, the vulture position at this time is calculated by the Formula (19).

(19) $$P_{i,\;\mathrm{new}\;}=rand\times P_{\mathrm{best}\;}+\omega \times x1(i)\times \left(P_{i}-c1\times P_{\mathrm{mean}\;}\right)+\;\omega \times y1(i)\times \left(P_{i}-c2\times P_{\mathrm{best}\;}\right)$$

### 3.4. MBESMBES-SVM Model

The improved vulture-search algorithm is used to optimize the kernel function and penalty factor in SVM. In the optimization process of the SVM model, MSE is used as the fitness function of MBES algorithm, which is defined in Equation (20) [19,35]

(20) $$fitness=\arg\min(MSE_{p})=\frac{1}{M}\sum_{n=1}^{M}{\left(y_{p}-y_{r}\right)}^{2}$$

where $y_{p}$ represents the predicted value, $y_{r}$ represents the measured value, and M is the total number of data samples.

The MBES–SVM algorithm uses MBES to automatically search for the optimal parameters of an SVR model. The BES algorithm performs iterations and updates the parameters based on the mean squared error (MSE) calculations on the test set. After a certain number of iterations, it finds a parameter configuration that yields the lowest MSE and highest predictive accuracy of the SVR model. The optimized SVR model with the selected parameters can then be used for new prediction tasks. 

The steps of the algorithm are as follows: 

Step 1: The data is divided into training and test sets.

Step 2: Define the Hyper-Parameter Space: Determine the hyper-parameters that need to be tuned and their respective ranges or discrete values.

Step 3: Set the control parameters of BES and initialize the Bald Eagle Population: Generate an initial population of bald eagles. Each bald eagle represents a candidate solution, which is a set of hyper-parameters.

Step 4: Evaluate Fitness: Evaluate the fitness of each bald eagle in the population. Fitness is determined by training and evaluating the model using the corresponding set of hyper-parameters. The fitness function should be defined by Equation (20).

Step 5: Update the position and speed of the bald eagle. Update the position (hyperparameter configuration) and speed (search direction) of each condor according to its current position, speed, and adaptability.

Step 6: Perform Exploration and Exploitation: The BES algorithm balances exploration and exploitation to search for better solutions. After the exploration and exploitation phases, reassess the fitness of the bald eagle and update it if any bald eagle achieves better performance.

Step 7: Repeat the Steps: Repeat steps 5 and 6 for a fixed number of iterations or until a termination criterion is met. The termination criterion can be a maximum number of iterations or reaching a desired level of performance.

Step 8: Extract the Best Solution: Once the algorithm terminates, extract the hyper-parameter set from the elite bald eagle, as it represents the best-performing solution obtained by the BES algorithm.

Step 9: Train and Evaluate the Model: Use the best hyper-parameter set obtained from the BES algorithm to train the model on the entire training dataset. Finally, evaluate the performance of the trained model on the test dataset or through cross-validation.

## 4. Experimental Analysis

### 4.1. Experimental Data

The data in this paper were obtained by SIDS mentioned in Section 2.2. The data include the ST (soil temperature), SMC (soil moisture content), and SEC (soil electrical conductivity), air humidity, rainfall, air humidity and light intensity of the tea plantation, which were measured from 20 August 2020 to 27 March 2021, with a total of 18,345 sets of data. After processing the data daily, 229 sets of data samples were generated and 216 sets of data were left after some abnormal data was clear. Some of the data are shown in Table 1.

### 4.2. Experimental Environment

The performance implementation of the algorithm was completed under Matlab 2014. Two experimental platforms were executed on a computer system operating under Windows 11. The computer’s CPU is an Intel(R) Core (TM) i7-9750H with a frequency of 2.60 GHz and 2.59 GHz, and a memory of 32 G.

The specific parameters of MBES-SVM, SVM, particle swarm optimization-support vector machines (PSO-SVM), and genetic algorithm-optimization support vector machine (GA-SVM) are shown in Table 2.

### 4.3. Evaluation Indicators

The model’s performance evaluation utilizes four distinct indicators, namely the root mean square error (MSE), root mean square error (RMSE), and R-square (R^2^). These factors can be calculated using Equations (21)–(23).

(21) $$MSE=\frac{1}{n}{\sum_{i=1}^{n}\left(y_{i}-{\hat{y}}_{i}\right)}^{2}$$

(22) $$\mathrm{RMSE}=\sqrt{\frac{1}{n}}\sum_{i=1}^{n}{\left(y_{i}-{\hat{y}}_{i}\right)}^{2}$$

(23) $$R^{2}=\frac{\sum_{i=1}^{n}\omega_{i}{\left({\hat{y}}_{i}-{\bar{y}}_{i}\right)}^{2}}{\sum_{i=1}^{n}\omega_{i}{\left(y_{i}-{\bar{y}}_{i}\right)}^{2}}$$

In the aforementioned equations, n represents the number of samples in a given dataset. The variable $\hat{y}$ indicates the predicted value, while $\overset{-}{y}$ represents the mean of the measured value of a particular feature. Finally, the variable y represents the measured value of a given feature.

### 4.4. Experimental Analysis

The initial 70% chronologically organized data points were designated as the training dataset, while the subsequent 30% data points were identified as the test dataset. The training dataset was then utilized to train the MBES-SVM network, SVM, PSO-SVM, and GA-SVM models. In PSO-SVM, each particle represents a candidate solution, and the optimal SVM model parameters are found by iteratively updating the position and velocity of the particles. In GA-SVM, the SVM model parameters are optimized through coding and genetic manipulation, such as crossover and mutation, and individuals with high fitness are selected to generate next-generation solutions. The MBES-SVM, PSO-SVM, and GA-SVM models differ in the optimization process. Differences in these algorithms can lead to different performances on different problems and datasets. Which model to use is depended on the specific application scenario and problem requirements.

Upon completion of training, the test dataset was applied to evaluate the performance of the mentioned model in order to generate the results illustrated in Table 3. To rigorously assess the performance of the model, repeated experiments are necessitated, of which the mean value is adopted as the conclusive outcome. 

As can be seen from the results in Table 3, MBES-SVM exhibits superior performance, with R2, MSE and RMSE of 0.8675, 0.0178 and 0.1333, SVM 0.8675, 0.0178, 0.1333, PSO-SVM 0.5662, 0.0711, 0.2666, and GA-SVM 0.5627, 0.0578 and 0.24.4, respectively. The PSO-SVM and GA-SVM algorithms are similar, SVMs are slightly better than both, and the MBES-SVM algorithm achieves the highest performance. The MBES-SVM model posited within this examination demonstrates the optimal predictive capacity, intimating that the model possesses profoundly robust prognostic ability, and the predictive efficacy is conclusively viable.

**Figure 2:**
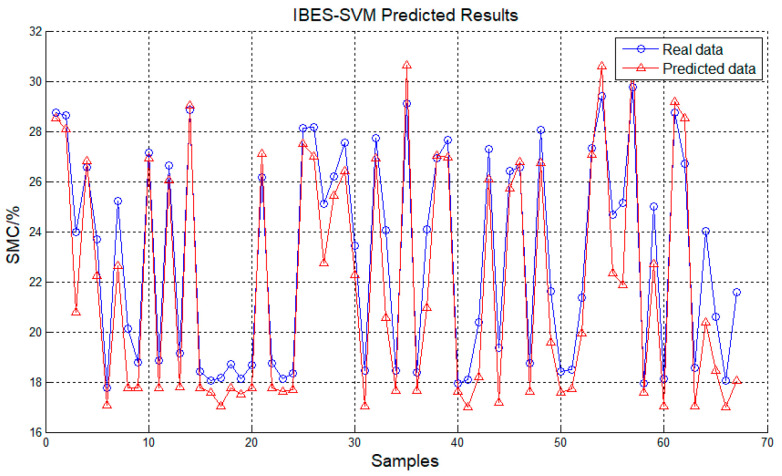
Outcome of the MBES-SVM Model.

**Figure 3:**
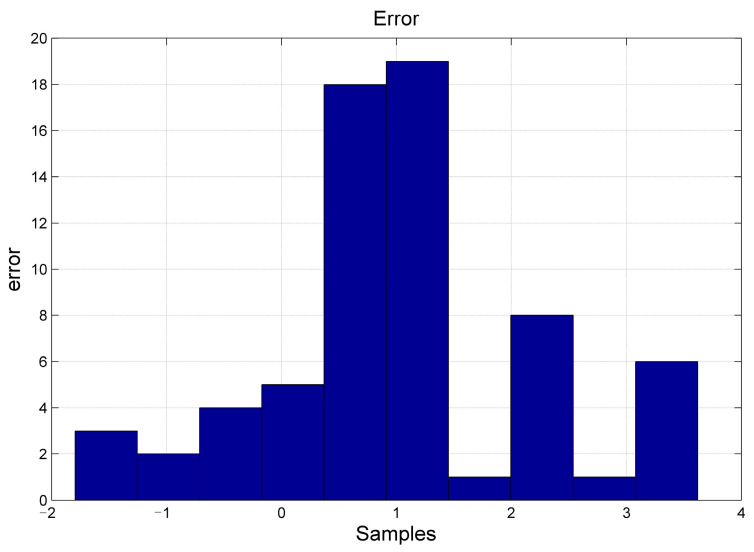
Error of the MBES-SVM Model.

The graphical illustrations in Figure 2 and Figure 3 delineate the outcomes of the prognostic test for the MBES-SVM model as well as the dispersal of prognostic errors in the MBES-SVM model test. From the figures, it is conspicuous that the anticipated values yielded by the MBES-SVM model are congruous with the authentic values, in which the margin of errors is regulated within ±0.2 with trivial deviations, thereby validating the viability and potency of this framework.

Using a very small number of sets as training and measurement sets, we conducted experiments and recorded the results. We randomly selected 10 sets of data and used 1, 2, 3, 4 and 5 data as the training set and the rest as the test set. The results are given in the following Table 4.

Although the training data is small, the prediction of the algorithm is still relatively satisfactory.

We did further experiments with a small training set to predict the effect of more test data. We randomly selected 216 sets of data and used 1, 2, 3, 4 and 5 data as the training set and the rest as the test set. The results are given in the following Table 5.

From the results of the experiment, the prediction is also satisfactory.

The MBES-SVM model proposed in this manuscript has the best prediction performance, which indicates that the model has good prediction ability, and the prediction performance is effective.

## 5. Conclusions

In summary, the study developed an improved SVM model by incorporating meteorological factors such as air temperature, relative humidity, rainfall, and strength of illumination as input, along with the traditional input parameters of soil properties such as soil temperature and soil electrical conductivity to predict the soil moisture content (SMC) in a tea plantation. The model was trained and tested using data from the tea plantation. Compared to other existing models, the improved model achieved a higher accuracy in SMC prediction with R^2^, MSE, and RMSE of 0.8675, 0.0178 and 0.1333, respectively.

The kernel function and penalty parameter embedded in the SVM model were fine-tuned by means of the bald eagle search algorithm; thereafter the enhanced MBES-SVM model was adopted for anticipating the soil moisture content in tea farms. The experiments substantiated that this model showcases sterling proficiency and exceptional generalization aptitude. It also investigated the viability of applying this model to forecast soil moisture content.

An advanced SVM technique for SMC modeling developed in this paper utilizes both soil properties and meteorological inputs. The improved model demonstrated promising potential for real-time SMC monitoring in tea plantations. Further enhancements in model parameterization and validation across plantations would solidify its practical utility as a comprehensive tool to support sustainable water resource planning for agriculture.

Although the model shows the best performance compared to previous approaches, there are also limitations for this research.

Limited Generalization: One limitation of the proposed SVM model may be its ability to generalize to different tea plantations or regions. It is important to investigate whether the model performs well across diverse geographical locations or if it is specific to a particular dataset. A follow-up test could involve evaluating the model’s performance on soil moisture data from multiple tea plantations across different regions.

Data Availability and Quality: The availability and quality of data can significantly impact the performance of machine learning models. It is important to assess whether the proposed model is sensitive to variations in data availability and quality. A follow-up test could involve evaluating the model’s performance using different datasets with varying levels of data availability and quality.

Feature Selection: The paper may mention the features used for predicting soil moisture content. It is important to investigate the impact of feature selection on the model’s performance. A follow-up test could involve exploring different combinations of features or using feature selection techniques to identify the most relevant variables for predicting soil moisture content in tea plantations.

Model Comparison: Comparing the proposed SVM model with other machine learning algorithms commonly used for soil moisture prediction can provide insights into its effectiveness. A follow-up test could involve comparing the SVM model with algorithms like Random Forest, Neural Networks, or Gaussian Processes to determine which model performs better in terms of accuracy and computational efficiency.

Robustness to Environmental Factors: Tea plantations are subject to various environmental factors such as rainfall, temperature, and humidity. It is essential to assess the robustness of the proposed model under different environmental conditions. A follow-up test could involve evaluating the model’s performance during different seasons or under varying weather conditions to determine its reliability and stability.

Long-Term Predictability: Tea plantations require long-term soil moisture predictions for effective irrigation management. It would be valuable to assess the long-term predictability of the proposed SVM model. A follow-up test could involve training the model on historical data and assessing its performance in predicting soil moisture content for future time periods.

Scalability: The scalability of the model is an important consideration, especially if it is intended for real-world applications. It would be beneficial to evaluate the model’s performance when trained on larger datasets or when predicting soil moisture content for larger tea plantation areas. A follow-up test could involve scaling up the dataset and assessing the model’s computational efficiency and prediction accuracy.

## Figures and Tables

**Table 1:** Partial raw data.

Air Temperature	Humidity	Light Intensity	Rainfall	ST	SMC	SEC	Time
21.85	100.00	1860.00	411.20	19.05	26.94	120.32	2021/3/27
15.70	100.00	134.00	537.60	18.87	26.88	118.56	2021/3/25
17.36	94.15	3492.50	503.45	17.63	26.89	118.76	2021/3/24
17.36	99.40	3888.29	536.37	17.53	27.06	119.16	2021/3/23
16.60	86.40	6658.00	537.60	16.98	27.18	120.27	2021/3/22
26.67	100.00	7702.67	409.60	21.14	27.06	116.31	2021/3/19
26.60	100.00	6266.00	409.60	20.67	27.10	116.17	2021/3/18
24.98	100.00	5482.75	410.00	19.75	27.15	116.15	2021/3/17
23.87	100.00	6968.00	420.93	18.77	27.24	115.75	2021/3/16

**Table 2:** Parameters of the experiment.

Modes	Item	Value	Modes	Item	Value
SVM	-c	50	BES	α	1.5
	-g	0.1		a	10
	-s	2		R	1.5
	-p	0.01		C1	2
PSO	c1	1.5		C2	2
	c2	1.7		N	100
	maxgen	200		Max_iter	100
	k	0.6		Dim	2
	wP	1	GA	maxgen	30
	v	3		sizepop	20
	popcmax	100		cbound	[0, 100]
	popcmin	0.1		gbound	[0, 100]
	popgmax	1000		v	5
	popgmin	0.01		ggap	0.9

**Table 3:** Evaluation of model.

Model	$R^{2}$	RMSE	MSE
SVM	0.8675	0.1333	0.0178
PSO-SVM	0.5662	0.2666	0.0711
GA-SVM	0.5627	0.2404	0.0578
MBES-SVM	0.9435	0.1392	0.0194

**Table 4:** Evaluation of model in smaller samples.

Number of Training Sets	MSE	R^2^
1	294.996	−3.35862 × 10^−16^
2	0.0625	0.6018
3	0.1088	0.8465
4	0.0560	0.7176
5	0.0106	0.9636

**Table 5:** Evaluation of model.

Number of Training Sets	MSE	R^2^
1	698.959	−2.28567 × 10^−16^
2	0.1168	0.0385
3	0.02984	0.7252
4	0.02299	0.8540
5	0.0058	0.9448

## Data Availability

Not applicable.
